# Examining linkages among multiple sustainable development outcomes: does the productive safety net program increase on-farm agrobiodiversity?

**DOI:** 10.1007/s10668-023-03257-2

**Published:** 2023-04-25

**Authors:** M. Kozicka, E. Gotor, T. Pagnani, M. Occelli, F. Caracciolo

**Affiliations:** 1 Bioversity International, Rome, Italy; 2 International Institute for Applied Systems Analysis, Laxenburg, Austria; 3 Department of Agricultural Sciences, University of Naples – Federico II, Naples, Italy; 4 Sant’ Anna School of Advanced Studies, Pisa, Italy; 5 School of Integrative Plant Science, Cornell University, Ithaca, NY USA

**Keywords:** PSNP, Ethiopia, Social protection program, Agrobiodiversity, On-farm labour, Q18, Q56

## Abstract

In Ethiopia, on-farm agrobiodiversity and the Productive Safety Net Program (PSNP) play a key role in building smallholders’ resilience. However, the impact of PSNP on on-farm agrobiodiversity is not yet well investigated. In this paper, we develop an analytical framework that links PSNP participation to on-farm agrobiodiversity. Both diverse farming systems and PSNP require labour inputs while providing income stabilization, which might result in a negative relationship between the two. Conversely, higher income from PSNP might allow farmers to increase their long-term on-farm investments, as opposed to the strategies oriented toward the highest immediate profit or calorie intake outcome. We base our empirical analysis on the World Bank’s Ethiopian Socioeconomic Survey, a panel dataset encompassing nearly 3000 respondents and a Tobit model, based on Difference-in-Difference and the Propensity-Score Matching methods. We find that Ethiopia’s PSNP has a negative impact on farm labour input, both in terms of labour intensity and duration. Furthermore, our results show that participation in the program is associated, on average, with lower on-farm crop diversity. We conclude that the PSNP participation may be crowding-out production stabilizing farming activities, such as intercropping or cover cropping, that are more labour intensive. Our findings call for embedding tools in the new phase of the PSNP (2021–2025) that could incentivise on-farm resilience-oriented investments, in particular leading to higher crop diversification.

## Introduction

Smallholders in low-income countries are particularly vulnerable to the negative impacts of climate change and other stressors, such as outbreaks of diseases and climate shocks. The diffusion of social safety nets and similar programs that target livelihood improvement of vulnerable populations is rapidly increasing, being currently ubiquitous in Africa (Bahru et al., 2020; Beegle et al., 2018). The United Nations (UN) 2030 Sustainable Development Goal (SDG) 1.3 recognizes the need for extensive coverage of the poor and the vulnerable by appropriate social protection programs. Even though their positive impact on resilience is well established (Godfrey-Wood & Flower, 2018), the mechanisms linking participation in the programs to various sustainable development outcomes, that constitute the resilience of socio-economic and ecological systems, are not yet well understood (Dyngeland et al., 2020). In this paper, we explore the links between a social protection program and on-farm agrobiodiversity, an inseparable element of sustainable development, and one of the SDG 15 components (Blicharska et al., 2019; FAO, 2018). We base our analysis on the case study of Ethiopia and its Productive Safety Net Program (PSNP) which aims at mitigating extreme poverty.

PSNP advocates for safeguarding a minimum level of food consumption and asset accumulation among vulnerable households. In such a paradigm, the relation between PSNP participation and farm household's agrobiodiversity is not well understood (Gotor et al., 2021) and it is not apparent. It might be positive, generating synergies through higher income and higher labour availability, leading to higher on-farm investments and eventually boosting agrobiodiversity at the farm household level. On the other hand, a trade-off narrative could emerge, assuming that higher and more stable income disincentives on-farm labour and crowds out agrobiodiversity as an alternative income- and consumption-stabilizing strategy. Existing evidence on the relationship between participation in social protection programs and on-farm crop diversification could support either of these hypotheses. This study aims to contribute to bridging this gap by investigating the relationship between on-farm agrobiodiversity cultivation and PSNP participation. The study adds to the body of literature that investigates the impact of participation in social protection programs on various sustainable development outcomes. To our knowledge, this is the first work assessing the effect of PSNP participation on on-farm agrobiodiversity.

The paper is structured as follows: Firstly, the authors describe the study background (Sect. 2) that includes the related literature (Sect. 2.1), the description of the PSNP in Ethiopia (Sect. 2.2), and an analytical framework of the relationships between PSNP enrollment, on-farm agrobiodiversity and labour (Sect. 2.3). Section 2 ends with the description of the study context (Sect. 2.4). The dataset used and the econometric models chosen are then explained (Sect. 3), while the results are presented in Sect. 4. Finally, Sect. 5 collects the discussions and policy implications of the findings.

## Study background

### Related literature

Benefits brought by higher levels of agrobiodiversity are well recognized. Agrobiodiversity improves pest suppression (Bommarco et al., 2013) and contributes to soil fertility (Tiemann et al., 2015). It further generates positive spill overs leading to improved ecosystems’ resilience (Yachi & Loreau, 1999). Moreover, on-farm crop diversification reduces consistently the risk of agricultural production failure (Davis & Schirmer, 1987) and market shocks, hence providing a form of insurance at the farm household level. Agrobiodiversity enhances productivity and yield stability, thus increasing and stabilizing households’ income (Bellon et al., 2020). Diversified farming systems also improve nutrition (Bellon et al., 2016) and reduce food insecurity (Beaglehole & Yach, 2003; Pagnani et al., 2021). Thus, higher crop diversity is key to building the resilience of smallholder farmers against climate change and crop disease outbreaks (Kozicka et al., 2020).

In order to increase levels of on-farm agrobiodiversity among vulnerable farm households, several ad-hoc interventions have emerged. There are programs directly focusing on agrobiodiversity cultivation (see, among others, the international community of practice ISSD—Africa Innovation for Seed Sector Transformation—established in 2012; Louwaars et al., 2013), as well as indirect interventions. Among the latter, national development strategies frequently implement protection programs to support livelihoods, mitigate income or food poverty, and enhance rural resilience (Dyngeland et al., 2020). Despite being multi-purpose initiatives, programs are often designed and evaluated as single interventions: Frequently, evaluations are mainly based on assessing whether the program’s primary objectives are met, while secondary outcomes, which are not core objectives, are rarely measured (Barrientos, 2012). This restricted focus augments the risk of ignoring trade-offs and negative externalities, potentially generating incomplete impact analysis on program effectiveness (Gehrke & Hartwig, 2018; Liao & Brown, 2018).

There is mounting evidence that farmers in low-income settings tend to use crop and animal diversity in response to experienced shocks, as income and consumption-stabilizing strategy (Hitayezu, 2016; Mulwa & Visser, 2020; Nguyen et al., 2017). There could hence emerge a negative externality of income or consumption-stabilizing social protection programs—by reducing risk exposure, they could crowd out on-farm diversity, which would have negative social and environmental consequences in the medium and long-terms.

In addressing this research gap, our study focuses on investigating links between the Ethiopian Productive Safety Net Program (PSNP) and agrobiodiversity levels.

### A description of the ethiopian productive safety net program (PSNP)

The Productive Safety Net Program (PSNP) is Ethiopia’s rural safety network for vulnerable smallholder households. These individuals are usually chronically food insecure, poor, and tend to be frequently affected by climate shocks. The program was started by the Government of Ethiopia in 2005 and operates in Afar, Amhara, Harare, Oromia, SNNP, Somali, Dire Dawa, and Tigray regions (MoARD, 2015). Currently, the program operates in the 282 most chronically food-insecure *woredas* (districts) in rural Ethiopia. The PSNP replaced an old system where aid depended on international emergency assistance, which resulted in unpredictable and volatile provisions (Jayne et al., 2002; Kehler, 2004). The PSNP aimed to provide certain transfers for a defined period (at least 5 years) (Bishop & Hilhorst, 2010). Indeed, PSNP offers direct support (cash or food) to almost 9 million poor people in Ethiopia, either by their participation in large-scale public works or by the means of unconditional transfers to those poor households with limited labour capacity (Hoddinott et al., 2012; Sharp et al., 2006). PSNP beneficiaries who are able to provide labour receive support for 6 months of the year. During this period, they received an average of 10 birrs (US$0.22) per day (data from 2010) to compensate for their engagement in the construction of community assets. Beneficiaries were employed for no more than 15 days per month (MoARD, 2015; Hoddinott et al., 2012). Most of the activities were scheduled between January and June so that they do not overlap with the agricultural works that mostly happen later in the year (Berhane et al., 2014). Roughly 15% of the participants in the program are incapable to provide labour, because of disability, illness or a very high household dependency ratio. These households receive free food or cash without a work requirement.

The fourth phase of PSNP (2015–2020) focused on climate change mitigation and adaptation by introducing microclimate management techniques such as terracing and small-scale irrigation. It has also introduced improvements to the planning and management of public works (Anderson & Farmer, 2015).

Since its establishment, the Ethiopian PSNP has proved to increase the resilience of the participants to adverse weather shocks and it contributed to self-reported increases in food security (Hailu & Amare, 2022; Knippenberg & Hoddinott, 2017). The study by Abay et al., (2020) indicates that PSNP beneficiaries were overall less affected by the compounded shocks that vulnerable households experienced in Ethiopia during the Covid-19 pandemic. Furthermore, numerous studies indicate that PSNP improves the beneficiaries’ food security (Berhane et al., 2014; Hoddinott et al., 2012), food consumption (Garcia & Moore, 2012), daily calorie intake per capita (Gilligan et al., 2013), and children’s nutritional status (Debela et al., 2015; Porter & Goyal, 2016). The program succeeded on these fronts, while it has failed on some other fronts, among others, on building households’ assets (Gilligan et al., 2009; Sabates-Wheeler et al., 2020).

PSNP, in its current form, does not directly encompass agrobiodiversity-enhancing initiatives, but the evidence shows that in programs targeting poor rural households, social benefits and environmental outcomes are frequently interconnected (Dyngeland et al., 2020). Indeed, even though PSNP focuses primarily on alleviating poverty in rural areas, the numerous interventions scope a wide range of social and environmental sub-targets: Among others, during the fourth phase of the PSNP (started in 2015), the goal of increasing resilience while improving environmental management led to establishing a biodiversity-enhancing intervention (Béné et al., 2012). Thus, on-farm agrobiodiversity could be considered a secondary but relevant target of the program. Indeed, Ethiopian smallholder farmers traditionally rely on agrobiodiversity to improve (among others) income stability, resilience, and food security (FAO, 2012; Michler & Josephson, 2017).

### Theoretical framework

It is well established that both agricultural interventions and social protection programs are effective, mutually reinforcing, tools for alleviating hunger and poverty among vulnerable smallholder households (Bellon et al., 2020; Tirivayi et al., 2016). Rural households in low-income countries are often affected by restricted access to resources, low productivity, disrupted markets, and they tend to be repeatedly exposed to combined and idiosyncratic shocks (Dorward et al., 2006). In absence of adequate insurance tools or risk-sharing mechanisms, these households might be forced to adopt sub-optimal agricultural strategies, such as farming the most profitable or the most calory-dense crops, which might be rewarding in the short-term but may generate negative agro-ecological trade-offs and, in the long-term, leading to lower farm productivity, poor nutrition and eventually higher susceptibility to shocks (Tirivayi et al., 2016). Social programs have the potential to reduce the vulnerability of rural households, by increasing their income and stabilizing consumption. Nevertheless, to ensure equity and durability of these effects, the programs need to result in building, protecting and deploying capital (human, social and natural) in an economically (Moroz, 2020) and environmentally sustainable manner (Bahru et al., 2020). As a result, programs that increase the sustainability of agricultural practices of their participants have a higher chance of improving their long-term resilience and eventual graduation out of poverty.

PSNP provides vulnerable households income support (conditional or unconditional), aiming to increase food consumption and build private assets while developing public infrastructure. These transfers lead to a higher and less volatile household income.

**Fig. 1:**
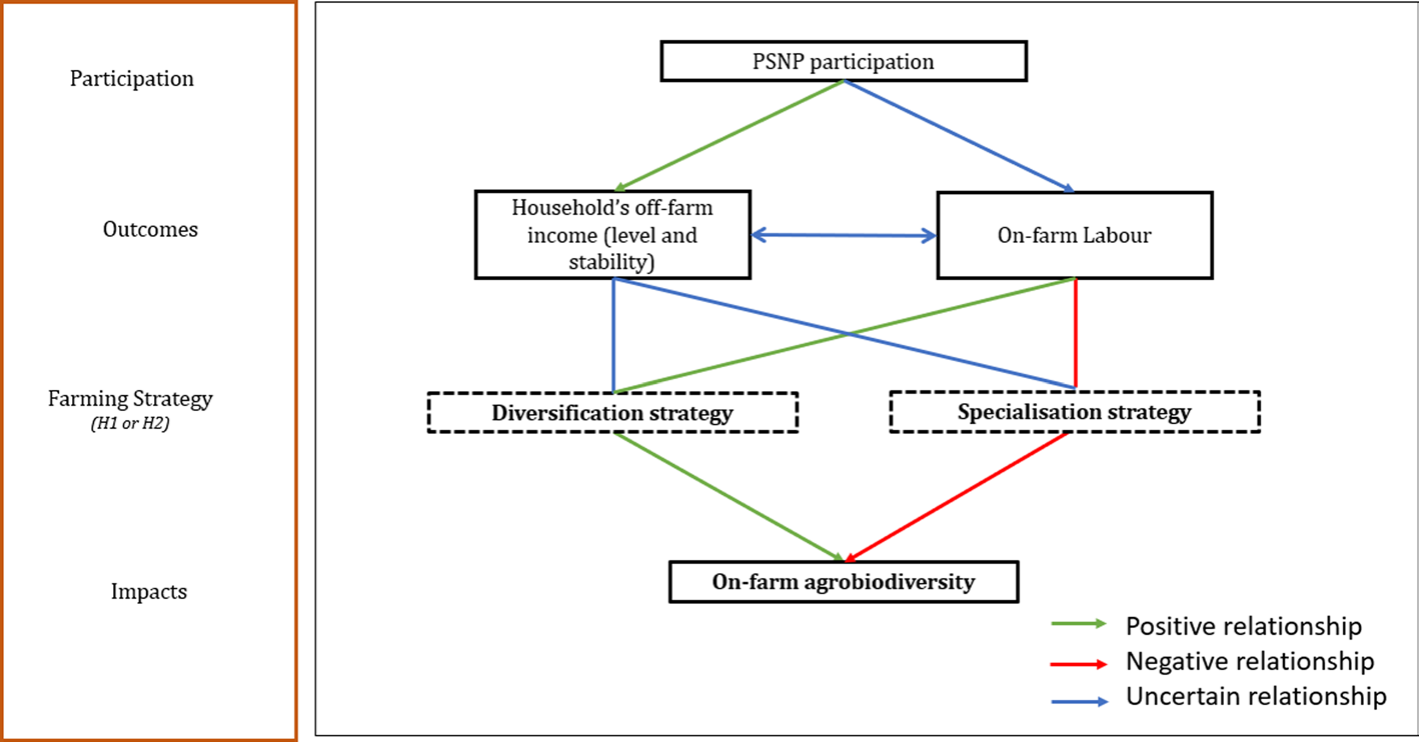
Theory of change: effect of PSNP on agrobiodiversity

We identify two impact pathways of the PSNP on agrobiodiversity (Fig. 1) (Gotor et al., 2021). The first one is through the income level and its volatility. Participation in the PSNP generates a stable inflow of cash and food—hence a positive relationship between PSNP participation and disposable household income (a green arrow in the framework). This income effect could support both farm diversification and specialization (indicated by blue lines in the framework). The reasoning is as follows: More stable income provided by PSNP can crowd out income- and consumption-stabilizing farming strategies toward riskier and more profitable monocropping systems (Kozicka et al., 2020). This specialization strategy would mean lower levels of agrobiodiversity as a result of the PSNP participation (red arrow in the framework). However, it could be also the case that by increasing household income, the participation shifts priorities from short-term food production increase of a more intensive farm focused on fewer crops, to longer-term benefits of a more resilient and stable production provided by a diverse system. In this case, the participation would result in a diversification strategy and consequently have a positive impact on the agrobiodiversity level (green arrow in the framework).

The second impact pathway is through on-farm labour. PSNP works are usually carried out during low farming seasons so that they do not disrupt households’ agricultural activities; however, they could have an indirect negative impact on farming intensity through the overall labour availability (Devereux et al., 2008). The relationship between PSNP participation and labour availability is not clear (blue arrow in the framework). Furthermore, the indirect impact of PSNP on on-farm labour through the increased income level and stability is not clear. It has been shown that households (limited-resource farmers and spouses) receiving income transfers chose to decrease their off-farm work (Vergara et al., 2004). This should mean that they either increase their leisure time or the on-farm labour. Consequently, the impact of the income on labour is not clear (blue line). Moreover, this relationship is two-way. Higher on-farm labour also influences household income. Diverse farming systems, for example highly diverse intercropping or field management systems, can be very labour intensive, while monocropping tends to require fewer labour inputs (Bisht et al., 2020). As a result, the linkage between the on-farm labour and any specialization strategy is negative (red arrow), while with any diversification strategy, it is positive (green arrow).

Finally, considering the impact of the strategy on agrobiodiversity levels is straightforward where a specialization strategy leads to lower on-farm biodiversity (red arrow), while a diversification strategy leads to higher on-farm biodiversity (green arrow).

Since the relationship between the PSNP participation and the on-farm labour is not known, while it is clearly positive between PSNP and income, in this study, we focus on the impact of PSNP on agrobiodiversity via the labour channel while controlling for income (on- and off-farm). It follows that we formulate the following hypotheses (H) on the net effect of participation in the program on farm labour and agrobiodiversity, which are stylized in the represented theory of change (Fig. 1):

#### H1

Positive impact (diversification strategy): PSNP participation leads to higher on-farm labour inputs and a net higher on-farm agrobiodiversity.

#### H2

Negative impact (specialization strategy): PSNP participation leads to lower on-farm labour inputs and a net lower on-farm agrobiodiversity.

### Study context

In Ethiopia, agriculture represents the mainstay of rural households in the country. Around 85% of Ethiopians work in the agricultural sector which is mainly subsistence, smallholder farming in nature.

The country is in the sub-tropical climatic zone, with one main rainy season (*meher*) in summer and a second shorter period of occasional rainfalls (*belg*) in the early spring. It is characterized by a highly diverse landscape. Agro-ecological systems in the highlands usually produce cereals (among others, wheat, barley, and teff) (Chamberlin & Schmidt, 2012). Cultivation of different crops on one plot is a common practice adopted by smallholder farmers to reduce vulnerability to both market- and climate-driven shocks (FAO, 2012; Michler & Josephson, 2017). As a result, Ethiopia is an important reservoir of agricultural biodiversity, including crop wild relatives (Egziabher, 1991). This valuable gene pool is, however, endangered among others due to land degradation, land use change, homogenization of agriculture, and increasing substitution of traditional with improved crop varieties.

## Materials and methods

### Data

**Fig. 2:**
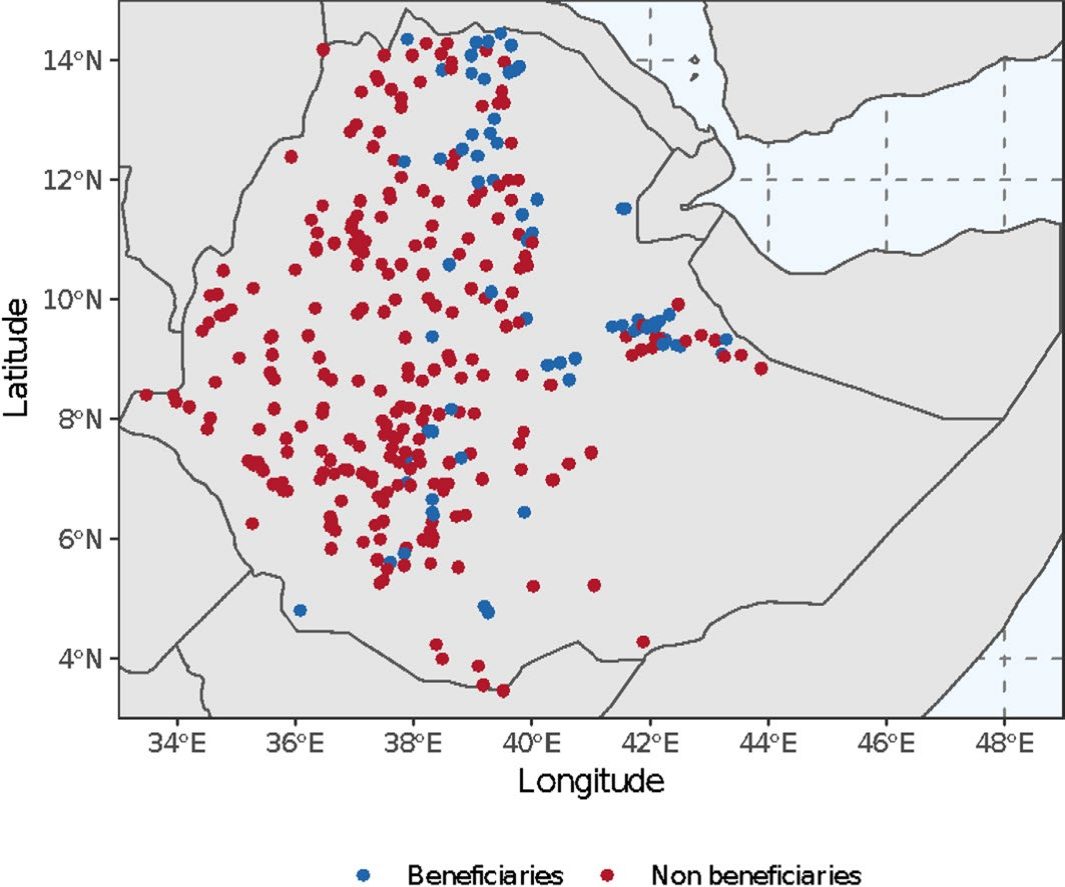
Map of Ethiopia’s PSNP as captured by the two waves considered by this study

For the analysis, we employed the Ethiopian Socioeconomic Survey (ESS), a household-level panel survey implemented every two years from 2011–2012. It is carried out jointly by the World Bank Living Standards Measurement Study-Integrated Surveys on Agriculture (LSMS-ISA) and the Central Statistics Agency of Ethiopia (CSA). The sample used for this analysis comprises 2,937 households, and it is drawn from all territories of Ethiopia (with the exception of the Sidama region) (Fig. 2). Respondents were surveyed in two rounds the first one—between 2011 and 2012, and the second one—between 2015 and 2016 (Table 1).

**Table 1:** Sample distribution

Region	Non-beneficiary households	Beneficiary households	Total
Tigray	172	98	270
Afar	5	27	32
Amhara	521	107	628
Oromia	612	28	640
Somali	82	38	120
Benshagul-Gumuz	114	0	114
SNNP	763	75	838
Gambela	91	0	91
Harari	83	15	98
Dire Dawa	14	92	106
Total	2,457	480	2,937
%	83.66	16.34	100.00

This study aims to assess the impact of PSNP on on-farm labour and agrobiodiversity during the specific timeframe under analysis. Consequently, we define a beneficiary as a household that has been a PSNP participant since the first wave of the panel, and a non-beneficiary as a household that was never a PSNP participant during the analyzed timeframe (Table 2). Households who participated in the program only in the first wave, but not in the following ones, were excluded from the analysis. Conversely, households that were not part of the program in the first wave, but did in the following ones, were considered beneficiary households. In that way, average changes over the four years due to PSNP participation can be identified by comparing beneficiaries to non-beneficiaries in the two waves. Approximately 16% of the households in our sample were categorized as beneficiaries. Even though the program is geographically ubiquitous, in three regions, Benishangul-Gumuz, Gambela and Sidama, there are no beneficiaries at all (Fig. 2 and Table 2). Robustness checks deleting these three regions led to estimates which are comparable for magnitude and significance with the one of the main econometric exercise (see the Appendix).

**Table 2:** Sampling strategy

Membership identified by this study	2011–2012	2015–2016
Non-beneficiary households	NP	NP
Excluded households	P	NP
Beneficiary households	NP	P
Beneficiary households	P	P

*With NP being non-participant in the PSNP program of that year and P being a participant in the PSNP program of that year*

### Methodology

To elicit the impact of Ethiopia’s safety net program on on-farm agrobiodiversity and, in general, on farming activities, two relevant outcomes have been selected: household labour supplied on-farm and on-farm agrobiodiversity levels. The first outcome was measured using two variables: *Farm Labour Total Days* and *Farm Labour Intensity*. *Farm Labour Total Days* are the total number of days of farm work done by the household members and individuals outside the household in the year under analysis. *Farm Labour Intensity* is an indicator given on the ratio of the working hours on the farm by household members to the total area of land cultivated.^1^ The first indicator (*Farm Labour Total Days*) provides a measure of the overall work done on the farm; however, it can be influenced, among other things, by the extent of the land. On the contrary, *Farm Labour Intensity* provides a comparable measure of farm labour as it is calculated by land area.

The second outcome of interest, on-farm agrobiodiversity was approximated using three complementary variables: the *crop richness index*, the *Simpson’s diversity index*, and the *Shannon index*. *Crop Richness* is the simple count of different crops cultivated by the household on the farm. The Simpson’s Diversity Index is based on the following formula:

1 $$\begin{array}{*{20}c} {{\text{Simpson's Diversity Index}} = 1 - \mathop \sum \limits_{j = 1}^{J} P_{j}^{2} } \\ \end{array}$$

where $P_{j} = A_{j} /\sum A_{j}$ is the proportion of the j-th crop area relative to the total area under cultivation. The value varies between zero (0) (only one variety cultivated) and 1. Finally, the Shannon index is based on the formula below:

2 $$\begin{array}{*{20}c} {{\text{Shannon Index}} = - \mathop \sum \limits_{j = 1}^{J} P_{j} \ln P_{j} } \\ \end{array}$$

Variables’ definitions and summary statistics for the entire sample are reported in Table 3.

**Table 3:** Variables’ definitions and summary statistics

Variables	Definitions	Mean(SD)
Gender HH head	1 if the household (HH) head is male, 0 otherwise	0.78 (0.42)
Age HH head	Age of the HH head in years	46.13 (15.17)
Education HH head	Grade completed by the HH head	5.11 (2.89)
Household size	Number of HH members	5.76 (2.48)
Dependency ratio	Ratio of productive versus unproductive HH members	1.38 (1.13)
Home assets index	Composite index constructed using factor analysis. Ranges from 0 to 100	21.11 (10.52)
Agriculture assets index	Composite index constructed using factor analysis. Ranges from 0 to 100	26.54 (16.01)
Extension program	1 if HH participated in extension programs, 0 otherwise	0.33 (0.47)
Other assistance	1 if HH received assistance other than PSNP, 0 otherwise	0.16 (0.37)
Climate shocks exposure	1 if the HH was exposed to climate shocks (such as drought, floods, landslides, and heavy rains), 0 otherwise	0.25 (0.43)
*Outcome variables*		
Farm labour total days	Days of farm work by both HH members and individuals outside of the HH	111.45 (151.56)
Farm labour intensity	Ratio of farm working hours by HH members versus total land cultivated	1144.38 (2260.70)
Crop richness	Number of crops cultivated	5.53 (3.93)
Simpson's index	Simpson's Diversity Index	0.48 (0.28)
Shannon index	Shannon Index	0.94 (0.60)

To infer empirically the effect of the PSNP participation on the selected outcomes and variables, the empirical analysis must account for two limitations: (1) the eligibility and assignment of PSNP are not a random process, and hence, there might be a selection bias; and (2) the variables selected in this analysis are strictly positive and zero censored. To address these empirical challenges, the methodology employed for identifying the impact of PSNP involved a Kernel propensity-score matching and a difference-in-difference estimator (DiD) implemented in a Tobit model.

The propensity score $p\left({Z}_{i}\right)$ was estimated using a probit model with the dependent variable coded as 1 for participant households and 0 for non-participants. More specifically, the conditional probability of participation (or propensity score) for each *i*-th household was estimated from the following participation model:

3 $$p\left( {Z_{i} } \right) = Prob\left( {G_{i} = 1} \right) = F\left( {Z_{i} ,b} \right) + e_{i}$$

where ${G}_{i}$ is a binary variable indicating whether the household is a beneficiary of the PSNP (${G}_{i}=1$ for the beneficiaries and 0 otherwise), and $b$ is the parameters vector measuring the influence of the observable household characteristics (*Z*) on being a beneficiary of the PSNP.

The estimate of propensity score provides the weights that will be used in the DiD models: The latter will be implemented to measure the impact of being a beneficiary of the PSNP on the outcomes of interest. To ensure maximum comparability of both groups and to account for eventual sources of inconsistency given by potential selection bias, the sample was restricted to the area of common support and each household was weighted by the probability of being a beneficiary of the PSNP (Guo & Fraser, 2014). The DiD Tobit model is specified as follows:

4 $$\begin{aligned} Y_{it}^{*} = & \beta_{0} + \beta_{1} X_{it} + \beta_{2} G_{i} + \beta_{3} D_{t} + u_{it} \\ Y_{it} = & \max (Y_{it}^{*} ,0) \\ X_{it} =\, & G_{i} *D_{t} \\ \end{aligned}$$

where ${Y}_{it}$ is the observed outcome and ${{Y}_{it}}^{*}$ is the latent variable with the normally distributed error term, ${u}_{it}$(0, *σ*^2^), ${D}_{t}$ is the binary variable that equals 0 in the first period and equals 1 in the second period, and ${X}_{it}$ is the interaction between ${G}_{i}$ and ${D}_{t}$. The estimate of the ${X}_{it}$ parameter (${\beta }_{1})$ provides the Kernel propensity-score matching DiD estimator.

The DiD estimator controls for time-invariant differences between the two household groups and allows to measure the average change in the outcome of the beneficiary households of the PSNP (i.e., treatment group) minus the average change in the outcome of the non-beneficiary households of the PSNP (i.e., control group). In equivalent terms, ${\beta }_{1}$ can be expressed as follows:

5 $$\begin{aligned} \hat{\beta }_{1}^{{\text{diff - in - diff}}} = & \left( {\overline{Y}^{{{\text{treatment}},{\text{ after}}}} - \overline{Y}^{{{\text{treatment}}, {\text{before}}}} } \right) - \left( {\overline{Y}^{{{\text{control}}, {\text{after}}}} - \overline{Y}^{{{\text{control}},{\text{ before}}}} } \right) \\ =\, & \Delta \overline{Y}^{{{\text{treatment}}}} - \Delta \overline{Y}^{{{\text{control}}}} \\ \end{aligned}$$

where $\Delta {\overline{Y} }^{\mathrm{treatment}}$ is the difference of the outcome variable measured at the second wave minus the initial value of the outcome measured at the first wave for the treatment group and $\Delta {\overline{Y} }^{\mathrm{control}}$ is the difference of the outcome variable for the control group. This empirical strategy provides a consistent and unbiased estimate of the impact of PSNP on agrobiodiversity and labour availability for the specific timeframe considered (from 2011 to 2015).

The variables employed to control against the sample selection bias refer to households' observable characteristics not influenced by the program, including household head characteristics (gender, age, and education), household size, dependency ratio, home and agricultural assets, farm size, shock exposure, participation in extension programs and receiving support other than from PSNP.

## Results

### Sample description

Table 4 reports differences in household characteristics and outcomes of interest for both household groups across both waves.

**Table 4:** Sample description

Variables	2011				2015			
	Non-Ben. HH	Beneficiary HH	*t*-test		Non-Ben. HH	Beneficiary HH	*t*-test	
Gender HH head	0.80	0.71	4.25	***	0.78	0.69	4.41	***
	(0.40)	(0.45)			(0.41)	(0.46)		
Age HH head	44.06	46.30	− 2.94	***	47.45	49.77	− 3.13	***
	(15.26)	(15.12)			(14.83)	(15.04)		
Education HH head	4.87	4.29	4.11	***	5.58	4.73	5.87	***
	(2.89)	(2.43)			(2.95)	(2.54)		
HH size	5.19	4.97	1.93	*	6.41	6.13	2.20	**
	(2.27)	(2.26)			(2.54)	(2.46)		
Dependency Ratio	1.18	1.20	− 0.36		1.56	1.69	− 2.21	**
	(0.97)	(1.01)			(1.20)	(1.37)		
Home Assets Index	29.54	29.26	0.84		12.80	12.36	1.49	
	(6.79)	(6.10)			(6.41)	(3.18)		
Agriculture Assets Index	41.12	40.99	0.45		12.11	11.37	2.01	**
	(5.92)	(5.92)			(7.30)	(7.39)		
Farm size	1.53	0.85	3.37	***	1.46	1.02	3.61	***
	(3.17)	(2.04)			(2.59)	(1.56)		
Extension Program	0.28	0.16	5.54	***	0.42	0.31	4.47	***
	(0.45)	(0.37)			(0.49)	(0.46)		
Other Assistance	0.11	0.19	− 4.79	***	0.13	0.62	− 27.16	***
	(0.31)	(0.39)			(0.33)	(0.49)		
Climate Shocks Exposure	0.17	0.23	− 3.27	***	0.26	0.67	− 18.85	***
	(0.38)	(0.42)			(0.44)	(0.47)		
*Outcome variables*								
Farm Labour Total Days	116.42	110.14	0.75		111.09	89.24	3.28	***
	(168.24)	(163.81)			(134.72)	(126.44)		
Farm Labour Intensity	1203.41	1344.61	− 1.15		1065.14	1047.63	0.17	
	(2495.69)	(2255.99)			(2012.91)	(2182.50)		
Crop Richness	5.62	3.97	8.23	***	5.99	4.22	9.57	***
	(4.18)	(3.13)			(3.83)	(3.00)		
Simpson Index	0.47	0.38	6.73	***	0.52	0.40	9.44	***
	(0.29)	(0.29)			(0.25)	(0.28)		
Shannon Index	0.95	0.73	6.97	***	1.02	0.76	9.41	***
	(0.62)	(0.60)			(0.56)	(0.56)		
*Number of observations*	2457	480			2457	480		

A HH refers to Households

Significance level: *p*-value < 0.01(***); < 0.05(**); < 0.10(*)

Compared to beneficiary heads of households, non-beneficiary heads of households under the PSNP are young males with a higher level of education. Significant differences exist between beneficiary and non-beneficiary households also in terms of the size of the land owned and participation in extension programs. Households benefiting from the PSNP have smaller amounts of land and lower levels of participation in extension programs; however, they have been more exposed to climate shocks and tend to receive other forms of assistance in addition to the PSNP. Interestingly, indexes of home assets and agriculture assets decreased significantly between 2011 and 2015 in both beneficiary and non-beneficiary households. We are unable to provide an explanation for this trend, but it represents an interesting aspect that could be investigated by further studies.

In terms of crop diversification, the difference between the two groups is statistically significant in both waves for all the selected indicators: crop richness, Simpson index, and Shannon index. Non-beneficiary households preserve higher levels of agricultural diversification on their farms than beneficiary households of the PSNP.

The differences between beneficiaries and non-beneficiaries of the safety net are less obvious with regard to the time devoted to agricultural activities. The difference in terms of Farm Labour Intensity is not significant between the two groups in either wave, while for Farm Labour Total Days, it is significant only in the second wave. On average, non-beneficiary households spent more days working on the farm.

### Econometric results

The purpose of this study is to increase the understanding of the relationship between the social safety net program and crop diversification. To this end, the impact that participation in the program has generated on two outcome variables of interest was analyzed: time devoted to agricultural activities and on-farm agrobiodiversity.

First, a participation model was used to calculate for each *i*-household the conditional probability of participation in the PSNP to control the source of inconsistency given by potential selection bias. Table 5 reports the estimate of the participation model. Age of household head, exposure to climate shocks, and receiving assistance other than that offered by the PSNP are all factors positively influencing participation in the Ethiopian safety net. Gender and education of household head as well as farm size and participation in extension programs negatively influence PSNP participation. Estimates by the participation model allow calculating the proper weighting scheme to rebalance and adjust the observations, removing self-selection bias.^2^

**Table 5:** Results of the propensity-score matching (PSM) method

Variable	Coef.	Std. Err.	*Z*	
Gender HH head	− 0.178	0.072	− 2.48	**
Age HH head	0.005	0.002	2.50	**
Education HH head	− 0.040	0.011	− 3.64	***
HH size	− 0.001	0.015	− 0.06	
Dependency Ratio	− 0.004	0.033	− 0.13	
Home Assets Index	− 0.002	0.005	− 0.36	
Agricultural Assets Index	− 0.001	0.005	− 0.16	
Farm size	− 0.055	0.017	− 3.13	***
Extension Services	− 0.280	0.072	− 3.89	***
Assistance	0.272	0.081	3.35	***
Climate Shocks Exposure	0.145	0.072	2.03	**
Constant	− 0.719	0.242	− 2.97	***

Significance level: *p*-value < 0.01(***); < 0.05(**); < 0.10(*)

Results of the DiD Tobit model reveal that, within the considered time span, the Ethiopian social protection program has a negative impact on household farm labour (Table 6). Households that benefited from PSNP transfers devote less time to agricultural activities on their farms than those who have not benefited from program transfers. This difference is reflected in the decrease in the total number of days of farm labour (around 28 days a year less or − 16.58%) and the farm labour intensity for the beneficiaries of the PSNP (− 11.53%). The effect is statistically significant. Moreover, on average, participation in the social protection program is linked to a lower diversity of on-farm crop cultivation. All three indicators of agrobiodiversity are lower for beneficiaries: DiD estimator is equal to − 0.40 for Crop Richness, − 0.05 for the Simpson’s Diversity Index, and − 0.10 for the Shannon Index. This indicates that participation in the PSNP results on average in 9.77% less crop richness, a 13.00% lower Simpson Index, and a 13.30% lower Shannon Index.

**Table 6:** Did results (2011–2015)

Outcome variables	Before			After			Diff-in-Diff	
	Control	Treated	Diff (T-C)	Control	Treated	Diff (T-C)		
Farm labour total days	81.75	98.42	16.67	94.06	82.31	− 11.74	− 28.42	**
Farm labour intensity *(log)*	5.05	5.48	0.43	5.80	5.57	− 0.22	− 0.65	***
Crop richness	4.74	3.67	− 1.07	5.60	4.13	− 1.47	− 0.40	*
Simpson index	0.40	0.33	− 0.07	0.48	0.36	− 0.12	− 0.05	**
Shannon index	0.78	0.63	− 0.15	0.94	0.69	− 0.25	− 0.10	**

Significance level: *p*-value < 0.01(***); < 0.05(**); < 0.10(*)

Results of a robustness check exercise, which confirms the sign and magnitude of the coefficients of the main model, are detailed in the Appendix (Table 7).

## Discussion

The PSNP is a large-scale social safety net program, the broad impacts of which have yet to be fully explored. This study tackles one of its possible unintended consequences and estimates the causal impact of the program on on-farm agrobiodiversity: The effect of PSNP on agrobiodiversity is here hypothesised to pass-through on-farm labour input and income stability. In doing so, we provide additional evidence on socio-economic as well as environmental co-existing impacts of one of the largest social protection programs in Africa.

We find a negative impact of the Ethiopian social protection program on both on-farm labour and agrobiodiversity. These results are in line with other recent studies on the impacts of the program, which found that, even though PSNP succeeded in improving consumption and food security (Gilligan et al., 2009), it has failed to foster asset building (Adimassu & Kessler, 2015). A study by Gilligan et al. (2009) found little impact of PSNP on asset building due in part to transfer levels that fell far below program targets.

Previous studies show similar results in other social protection programs. For example, Devereux (2006) found that the cash transfer program in South Africa had limited effects on on-farm investment with respect to the purchase of inputs. According to the study, carried out in 2006, only 3.4% of the participants used cash transfers to buy fertilizer, while only 11.5% purchased seeds.

Additionally, the demand for labour in public works could also yield what is commonly defined as the “crowding-out” effect (Andersson et al., 2011): Even though the PSNP is designed to avoid public works provision during any farming seasons, evidence shows that this might not be observed in all the districts covered by the program (Devereux et al., 2008). This problem was especially reported in Chiro, Fedis Kalu, Kilte Awlalo and Lasta *woredas*, where there was a direct overlap in the timing between the farming season and the delivery of public works.

Furthermore, it is possible that PSNP prematurely graduates many households, while there is no evidence of livelihood strengthening (Sabates-Wheeler et al., 2020).^3^ Given the projected high exposure to climate change shocks of rural households in Sub-Saharan Africa, these outcomes raise concerns about the capacity of the poor farm households to absorb any upcoming shocks, especially those that will graduate from the program. Furthermore, the extent to which the PSNP affects household income diversification strategies beyond agriculture remains uncertain (Conway & Schipper, 2011; Davies et al., 2009). This requires further investigations.

Our findings strongly suggest little coordination in PSNP between short-term socio-economic needs and long-term agroecological objectives, in line with the findings of Tirivayi et al. (2016). Coupling PSNP, a social protection program, with agricultural interventions could offer significant co-benefits in the form of better farm outcomes and improved farm household resilience. For example, Hoddinott and co-authors (2012) showed that households who participated for five years in PSNP and, at the same time received other agricultural transfers, specifically the OFSP/HABP Household Asset Building Program, had significantly higher agricultural yields than OFSP/HABP participants alone. Participants in only PSNP had no advantage in the increased agricultural input use or productivity. These effects are important to consider not only for immediate food consumption and income benefits but also for building resilience and food security in the long-term.

The complexity of the Sustainable Development Agenda—entering its last decade—calls for a careful planning of the next phases of existing Sub-Saharan social protection programs, PSNP *in primis*, with careful consideration of their secondary effects and trade-offs they generate between various objectives. Additional income from the participation in PSNP, with adequate perspectives on how to re-invest it in the farm, could be used to improve the farm assets, building resilience and eventually graduating the participants from poverty. In fact, PSNP could meet two objectives: improved food security in the short-term; and in the long-term promote on-farm investment, given the increasing severity and frequency of climate shocks and other stressors. The trade-offs can be addressed by including specific interventions and trainings on the return to rural investments that favor reinvestment in farming activities; benefits of agricultural biodiversity, and asset building—in the short and the long-term. These interventions should bolster resilience, food security, and livelihoods also in the long run.

This study is not exempt from limitations. Primarily, our theoretical model assumes the existence of a positive relation between PSNP and income, and we analyzed only the impact of PSNP on agrobiodiversity via the labour channel. For this reason, our estimates of the impact on agrobiodiversity can be considered conservative, even if the empirical model controlled for income (on- and off-farm) in the participation model. Further research could address this issue by examining explicitly also the income stability/volatility channel. A further limitation of the present study is that it only controls for sample selection bias, while the estimates may still suffer from omitted variable bias, which is another potential source of endogeneity. We attempted to minimize this relevant issue by including in the analysis several households’ characteristics while the presence of unobserved heterogeneity (hidden bias) was formally tested using the Rosenbaum bounds strategy (Rosenbaum, 2002).

## Conclusions

This paper provides evidence that PSNP significantly reduces on-farm household labour input and agrobiodiversity cultivation of its beneficiary households. The effect is robust across all crop diversity and farm labour indicators employed.

The findings and insights of this study could be useful in guiding additional measures in the Ethiopian social protection program, especially as the program moves into its fifth operational phase. While the Fourth Phase of PSNP focused more on climate-centred actions, this fifth phase should be oriented toward improving the participants’ resilience. It follows that the new PSNP phase should prioritize training farmers on the importance of rural investments. To achieve its goal, Phase V should incorporate or strengthen resilience-oriented approaches, with actions that instill higher awareness among farmers about the importance of adequate investments in their farm enterprises in order to improve food security and strengthen livelihoods.

Additionally, given the negative relationship observed between on-farm labour and participation in the social safety net program, there is a need for parallel evaluation programs aimed at understanding participants’ challenges and needs in maintaining their engagement and investments in their farms. Finally, in the face of the need to simultaneously address the increasing frequency and severity of climate change-related shocks and the global biodiversity crisis, we emphasize the need to take action and include agrobiodiversity outcomes into consideration when designing the new phase of PSNP.

## Data Availability

Data for this study are freely available and accessible in the World Bank Microdata Library (https://microdata.worldbank.org/index.php/home), section related to the Ethiopian Socioeconomic Survey (https://microdata.worldbank.org/index.php/catalog/lsms).

## Appendix

See Table

**Table 7:** Robustness check exercise (first table PSM score, second table Tobit model coefficients)

Variable	Coef.	Std. Err.	*Z*	
Gender HH head	− 0.195	0.074	− 2.65	***
Age HH head	0.004	0.002	2.20	**
Education HH head	− 0.039	0.011	− 3.48	***
HH size	0.001	0.015	0.09	
Dependency Ratio	− 0.010	0.033	− 0.28	
Home Assets Index	− 0.002	0.005	− 0.38	
Agricultural Assets Index	− 0.001	0.005	− 0.22	
Farm size	− 0.059	0.018	− 3.33	***
Extension Services	− 0.329	0.072	− 4.54	***
Assistance	0.264	0.083	3.20	***
Climate Shocks Exposure	0.091	0.072	1.26	
Constant	− 0.595	0.248	− 2.40	**

Outcome variables	Before			After			Diff-in-diff	
	Control	Treated	Diff (T-C)	Control	Treated	Diff (T-C)		
Farm labour total days	82.63	98.40	15.77	93.37	81.97	− 11.40	− 27.17	**
Farm labour intensity	5.03	5.48	0.45	5.77	5.57	− 0.20	− 0.65	***
Crop richness	4.78	3.66	− 1.12	5.58	4.12	− 1.46	− 0.34	
Simpson's index	0.40	0.33	− 0.07	0.49	0.36	− 0.12	− 0.05	**
Shannon index	0.79	0.63	− 0.16	0.95	0.69	− 0.26	− 0.10	**

The sample employed in this robustness analysis is drawn from all territories of Ethiopia, except those where no beneficiaries are registered (i.e., Benishangul-Gumuz, Gambela, and Sidama regions), and it includes 2960 households. As it is possible to observe, the results of the PSM and DiD model are unchanged

Significance level: *p*-value < 0.01(***); < 0.05(**); < 0.10(*)

7.

## Footnotes

1 The “Farm Labour Intensity” variable was included in the model as a logarithm since the uncensored part of the variable is distributed according to a log-normal distribution.

2 Balancing property (*p* < 0.10) was satisfied following Becker and Ichino (2002) strategy. It implies that both the average propensity score and the average for each explanatory variable between treated and untreated households must be equal.

3 Graduation is a common framework for thinking about how vulnerable households can move off programs of livelihood support into resilient and self-reliant, market-based living (refer to Sabates-Wheeler et al. 2020 for additional details on the framework and the related outcomes). Premature graduation entails the fact that participants complete their enrollment into PSNP and exit the assistance program. For PSNP participants, often this happened despite the lack of evidence on livelihood strengthening (Sabates-Wheeler et al. 2020). PSNP evaluations proved that an initial 3-years graduation trajectory was overly ambitious. Nonetheless, tweaks to the program's design, further aimed to accelerate graduation (Sabates-Wheeler et al. 2020).
